# Expression of PON1, PON2, PON3 and MPO Genes in Patients with Depressive Disorders

**DOI:** 10.3390/jcm11123321

**Published:** 2022-06-09

**Authors:** Katarzyna Bliźniewska-Kowalska, Piotr Gałecki, Kuan-Pin Su, Angelos Halaris, Janusz Szemraj, Małgorzata Gałecka

**Affiliations:** 1 Department of Adult Psychiatry, Medical University of Lodz, 91-229 Lodz, Poland; katarzyna.blizniewska-kowalska@umed.lodz.pl (K.B.-K.); piotr.galecki@umed.lodz.pl (P.G.); 2 College of Medicine, China Medical University, Taichung 404, Taiwan; cobol@cmu.edu.tw; 3 An-Nan Hospital, China Medical University, Tainan 709, Taiwan; 4 Department of Psychiatry and Behavioral Neuroscience, Stritch School of Medicine, Loyola University Chicago and Loyola University Medical Center, 2160 South First Avenue, Maywood, IL 60153, USA; ahalaris@luc.edu; 5 Department of Medical Biochemistry, Medical University of Lodz, 92-215 Lodz, Poland; janusz.szemraj@umed.lodz.pl; 6 Department of Psychotherapy, Medical University of Lodz, 91-229 Lodz, Poland

**Keywords:** depression, paraoxonase, myeloperoxidase

## Abstract

**Background:** Taking into account the role of oxidative stress in neurodegeneration, we sought to evaluate the expression of genes for select enzymes with antioxidant properties (paraoxonases PON1, PON2 and PON3 and myeloperoxidase MPO) at the mRNA and protein levels in patients with depressive disorders. We further sought to determine the impact of oxidative stress in the etiopathogenesis of this group of mood disorders. **Methods:** A total of 290 subjects (190 depressed patients, 100 healthy controls) took part in the study. Sociodemographic and clinical data were collected. The severity of depressive symptoms was assessed using the Hamilton Depression Rating Scale (HDRS). Venous blood was collected. RT-PCR was used to assess gene expression at the mRNA level, while enzyme-linked immunosorbent assay (ELISA) was used to assess gene expression at the protein level. **Results:** The expression of the PON2 and PON3 genes at the protein level was significantly higher in depressive patients than in healthy controls. mRNA expression of the PON1, PON2 and PON3 genes was slightly higher in patients with depressive disorders than in the control group, however, this relationship was not statistically significant. On the other hand, the expression of the MPO gene at both mRNA and protein levels was significantly lower in patients with depressive disorder than in the control group. **Conclusions:** Our results are not in agreement with many studies on enzymes involved in maintaining oxidative balance. Our findings may not support the utility of paraoxonases (PON) or myeloperoxidase (MPO) as promising biomarker candidates of depression pending larger and well controlled studies.

## 1. Introduction

Oxidative stress (OS) is an imbalance between pro-oxidative processes (the harmful effects of reactive oxygen species (ROS)) and antioxidant processes (that is, the body’s ability to neutralize free radicals and repair the damage they cause) [1]. Over-production of ROS and depletion of antioxidant defenses trigger pro-inflammatory signaling, damaging cells and leading to their apoptosis. Failure to maintain redox homeostasis by cells and the consequent production of pro-inflammatory mediators lead to cell necrosis. The central nervous system (CNS) is particularly susceptible to oxidative stress (OS) due to its higher oxygen consumption, higher lipid content and weaker antioxidant defense. OS is the main cause of neurodegeneration [2].

Research studies confirm that depressive disorders are accompanied by a decreased antioxidant state and the induction of oxidative and nitration pathways (O&NS). These studies hypothesize that aberrations in the O&NS pathways are—along with inflammatory processes—key components of depression pathogenesis [3,4,5]. Therefore, while searching for reliable biological markers of depression, we also considered factors influencing the oxidative balance of the organism, such as paraoxonases (PON) and myeloperoxidase (MPO).

Paraoxonase (PON) is an enzyme involved in the hydrolysis of ester bonds in the body. However, growing interest in this molecule is mainly related to its antioxidant activity. This enzyme occurs in three isoforms: PON1, PON2 and PON3, and PON genes are located on the long arm of chromosome 7 and show almost 70% similarity in terms of chemical structure. The best-known isoenzyme is PON1, a protein synthesized in the liver and in small amounts also in epithelial tissue. PON1 is closely related to the high-density lipoprotein (HDL) fraction. It protects low-density lipoproteins (LDL) against oxidation and is involved in the metabolism of toxins and drugs [6,7,8].

Another molecule we studied is myeloperoxidase (MPO), a heme-containing peroxidase expressed mainly in neutrophils. MPO catalyzes the formation of reactive oxygen intermediates that play an important role in microbial killing by neutrophils. Furthermore, it is a local mediator of inflammation in various inflammatory diseases. MPO deficiency results in intensification of the inflammatory response, and it affects neutrophil functions including cytokine production [9,10]. Taking into account its importance in inflammatory processes and in maintaining oxidative balance, we hypothesized that the expression of the gene for MPO may also play a role in the etiopathogenesis of depression.

The aim of this study was to evaluate the expression of genes for selected enzymes with antioxidant properties (paraoxonases PON1, PON2 and PON3 and myeloperoxidase MPO) at the mRNA and protein levels in patients with depressive disorders and to determine their impact in the etiopathogenesis of depression.

## 2. Methods

### 2.1. Characteristics of Study Participants

A total of 290 subjects took part in the study. The study group included 190 patients (117 female, 73 male) hospitalized due to the diagnosis of a depressive episode or recurrent depression disorder (rDD) (32 female and 33 female, respectively), according to the ICD-10 criteria [11]. The control group consisted of 100 healthy volunteers (66 female, 34 male) with a negative history of a mental disorder and no depressive symptoms. There was no statistically significant difference between the patient and the control group in regards to gender (*p* = 0.4583). The mental state of all participants was assessed by a psychiatrist on the day of acceptance to the study. The exclusion criteria were as follows: a psychiatric diagnosis other than depressive disorders, serious neurological or somatic diseases (including autoimmune diseases, current acute infection) that could affect the expression of PON1, PON2, PON3 and MPO, and abuse of or addiction to psychoactive substances. Individuals taking part in the study were native to Poland. Participation in the study was voluntary. Written informed consent was obtained from each subject according to the study protocol that had been approved by the Bioethical Committee of the Medical University of Lodz (No. RNN/833/11/KB).

Demographic data were obtained from all study participants. Data concerning the course of the depressive disorder were collected using the Composite International Diagnostic Interview (CIDI) [12], also taking into account the duration of the disease (in years), number of depressive episodes and number of psychiatric hospitalizations. The severity of depressive symptoms was assessed using the Polish adaptation of the 17-item Hamilton Depression Rating Scale (HDRS) [13]. Cronbach’s alpha (tau-equivalent reliability) for this scale was 0.70; the sensitivity coefficient was 0.78 and the test relevance coefficient was 0.75 [13,14]. Hospitalized patients were enrolled in our study; hence, the majority of them were in a moderately severe (HDRS score 18–29) or severe (HDRS score > 30) depressive episode (mean = 22.82 +/− 6.86). For the study group of depressed patients blood was collected at the beginning of the hospitalization, when the depressive symptoms were the most severe and before the modification of existing antidepressant treatment. Table 1 illustrates the demographic and clinical characteristics of both study groups.

### 2.2. Molecular Analysis

Peripheral venous blood samples were collected from all the participants. RT-PCR was used to assess PON1, PON2, PON3 and MPO gene expression at the mRNA level, while ELISA was used to assess their expression at the protein level.

#### 2.2.1. mRNA Gene Expression Assessment

Total RNA isolation from the patients’ peripheral blood lymphocytes was performed using InviTrap Spin Universal RNA Kit (Stratec Molecular, Berlin, Germany) based on the manufacturer’s recommendations. The quantity and purity of isolated RNA was spectrophotometrically estimated (Picodrop -VWR International Corporate LLC, Radnor, PA, USA). The quality of samples was assessed using an Agilent RNA 6000 Nano Kit on a 2100 Bioanalyzer (Agilent Technologies–Santa Clara, CA, USA) in accordance with the producer’s guidance. Electrophoretogram and RIN values were used to determine the level of degradation of total RNA. Only samples with RIN value >7 were subject to further analysis. Isolated RNA was stored at −70 °C.

Reverse Transcription reaction was carried out using a TaqMan^®^ RNA Reverse Transcription Kit based on the manufacturer’s recommendations, using specific Hs00166557_m1, Hs 00165563_m1, Hs01023629_m1, Hs 99999196_m1 and Hs04194366_g1 probes for PON1, PON2, PON3, MPO and RPL13A genes, respectively, delivered using Applied Biosystems (Foster City, CA, USA). The samples were incubated (30 min, 16 °C and 30 min, 42 °C) in a thermocycler (Biometra, Göttingen, Germany). Reverse transcriptase was inactivated (5 min, 85 °C) and the obtained cDNA was stored at 20 °C.

Real-Time PCR reaction was conducted using TaqMan^®^ Universal PCR Master Mix, No UNG (Applied Biosystems, Foster City, CA, USA) according to the protocol provided by the manufacturer. The Ct comparative method was used to calculate the relative expression of mRNA of the studied genes [15]. The level of PON1, PON2, PON3 and MPO gene expression in particular tissues was normalized in relation to the RPL13A reference gene. Each target probe was amplified in a separate 96-well plate. All samples were incubated at 50 °C for 2 min and at 95 °C for 10 min and then cycled at 95 °C for 30 s, at 60 °C for 30 s and at 72 °C for 1 min; 40 cycles were performed in total. Fluorescence emission data were captured and mRNA levels were quantified using the critical threshold (Ct) value. Analyses were performed using ABI Prism 7900 HT (SDS Software) [16].

#### 2.2.2. Protein Expression Assessment

Serum total protein concentration and the analytical curve for serum albumin were determined. Both the examined samples and the reference samples were run in parallel in three repetitions. Sample absorbance was measured using Multiskan Ascent Microplate Photometer (Thermo Labsystems, Philadelphia, PA, USA) at λ = 562 nm and total protein concentration was calculated from the standard curve equation. The serum concentration of PON1, PON2, PON3 and MPO proteins was determined using the Human PON1, PON2, PON3 and MPO ELISA Kit (MyBiosource San Diego CA, USA) according to the protocols provided by the manufacturer. β-actin was used for endogenous control of protein concentration in the samples and determined with the help of the Human Actin Beta (ACTb) ELISA Kit (BMASSAY) based on the manufacturer’s recommendations. The absorbance of the samples was measured using a Multiskan Ascent Microplate Photometer (Thermo Labsystems, Philadelphia, PA, USA) at λ = 450 nm. Analytical curves were constructed for the analyzed proteins to determine the protein concentration.

### 2.3. Statistical Analysis

A chi-squared test was used for contingency cross-tables. Generalized linear models (GLM) with robust standard errors were performed to test differences in numerical traits between the studied groups. All models controlled for the participants’ age and gender. The gene expression levels were log transformed before testing the hypothesis. Pearson correlation coefficients for variables measured on the same scale and Spearman rank correlation coefficients for traits measured on various scales were computed. A level of *p* < 0.05 was deemed statistically significant. All statistical procedures were set as two-tailed. The statistical analysis was carried out using IBM SPSS Statistics, v. 28 (IBM Corporation, Armonk, NY, USA).

## 3. Results

### 3.1. mRNA Expression

mRNA expression of PON1, PON2 and PON3 genes was slightly higher in patients with depressive disorders than in the control group, however this relationship was not statistically significant (PON1 *p* = 0.5895, PON2 *p* = 0.1342 and PON3 *p* = 0.2818) (Table 2). On the other hand, the expression of the MPO gene at the mRNA level was significantly lower in patients with depressive disorders than in the control group (*p* = 0.0014) (Table 2).

### 3.2. Protein Expression

Expression of PON family genes (PON1, PON2 and PON3) at the protein level was higher in depressive patients than in healthy controls, but this relationship was significant only in the case of PON2 and PON3 (PON1 *p* = 0.2274; PON2 *p* < 0.0001; PON3 *p* = 0.0363) (Table 3). Protein expression of the MPO gene was significantly lower in patients with depression compared to healthy individuals (*p* = 0.0006) (Table 3).

### 3.3. Correlation with Clinical Variables

In the group of depressed patients, we computed the correlation between mRNA and protein expression for selected genes and clinical variables, including the severity of depressive symptoms as measured by the HDRS score, the number of hospitalizations, disease duration (in years) and the number of depressive episodes. There was no statistically significant relationship between HDRS scores, disease duration or number of depressive episodes, and the expression at the mRNA level or at the protein level for any of the analyzed genes. The only statistically significant association observed was between PON2 expression (both at mRNA and protein levels) and the number of hospitalizations (correlation coefficients 0.210, *p* = 0.0040 and 0.202, *p* = 0.0057, respectively), meaning the higher number of hospitalizations, the higher the expression of PON2 (Table 4).

### 3.4. Correlation with Age

Although the study patients were significantly older than the controls, no statistically significant correlation was observed between age and expression of any of the analyzed genes in either group (Table 5).

## 4. Discussion

As previously mentioned, the family of human paraoxonase genes has three members: PON1, PON2 and PON3. The products of these three genes are the enzymes paraoxonase 1 (PON1), paraoxonase 2 (PON2) and paraoxonase 3 (PON3) [17]. The name of this enzyme family is derived from the ability of PON1, the most studied isoform, to hydrolyze the pesticide paraoxon [18,19]. However, PON1 is not the oldest paraoxonase. From the evolutionary point of view, based on structure homology, PON2 appears to be the oldest member of this family, followed by PON3 and then PON1 [17,20]. PON1 and PON3 are mainly synthetized in the liver, and then secreted into the blood where they primarily bind to high density lipoproteins (HDL), whereas PON2 is ubiquitously expressed in all different kinds of tissues [17,18,19]. The studies emphasize that plasma activity, especially of paraoxonase 1, is substrate-dependent, but also dependent on gene single nucleotide polymorphisms (SNP’s) [19]. PON1 is polymorphic and the best-known polymorphism of this gene is Q192R, influencing the substrate-dependent catalytic PON1 activity [19,21]. Another important SNP is L55M, although it does not affect PON1 catalytic activity, but is associated with its plasma protein level (PON1M55 with low protein level) [19,22]. Based on our knowledge about limitations in assessing enzyme activity, we studied the expression of three paraoxonases genes (PON1, PON2 and PON3) both at the mRNA and protein levels.

There appears to be agreement that PON1 activity is altered in patients with depressive disorders [19]. However, Sarandol et al., in their study in which they enrolled 86 depressed patients and 36 healthy controls, reported no association between depressive disorders and PON1 activity (either POase or AREase PON1 activities). They also suggested that antidepressant treatment may reduce plasma paraoxidase activity and that lipoprotein oxidation appears to be increased in depressed patients [23]. Similarly, Kodydkova et al. investigated 35 drug-naïve depressed women and showed normal AREase PON1 activity. However, the activity of other main antioxidative enzymes was altered and the study group was characterized by increased oxidative stress [24]. Barim et al. examined patients with depression before and after three months of therapy with citalopram at a dose of 40 mg/day. They reported lower levels of paraoxonase arylesterase (AREase) in depressive patients before treatment (restored after therapy) than in the control group [25]. Kotan et al. [26] and Bortolasci et al. [27] came to similar conclusions. The latter suggested that lowered plasma PON1 activity is a trait marker of major depression [27]. They also assumed that the interaction between external factors (smoking) and genetic factors (Q192R polymorphism of the PON1 gene) also influenced the chance of developing depression [27]. A meta-analysis [28] taking into account the studies described above highlights the fact that serum levels of paraoxonase and antioxidants are lower and the levels of free radicals and oxidative damage products are higher in patients with depression than in control groups. The levels of antioxidants increased and the levels of oxidative damage products decreased after anti-depressant pharmacotherapy [28]. Moreira et al. stated that lowered PON1 activities are strongly associated with depression, recurrence of depressive disorders, increased disability and lowered quality of life [29]. They examined 32 major depressed patients (MDD) and showed significantly lower PON1 activities in this group of patients, which is partly related to the number of previous depressive episodes [29]. The results concerning the activity of paraoxinase in depression seem to be less clear-cut. Ullas Kamath et al., in their study of 24 depressed patients, found significantly greater PON1 serum levels in patients with moderate depression than in controls [30]. Although our research relates to gene expression, it appears to be in line with the findings of Ullas-Kamath et al. We reported slightly higher (but not statistically significant) mRNA expression of PON1, PON2 and PON3 genes in patients with depressive disorders than in the control group. Expression of PON family genes at the protein level was higher in depressive patients than in healthy controls, but this relationship was significant only in the case of PON2 and PON3 and not of PON1. On the other hand, Ogłodek [31] tested a large number of patients with MDD and PTSD (in total 460 patients, only 60 with PTSD alone) and concluded that depression became more severe at decreased PON-1 concentrations (also measured with an ELISA test) [31]. In our study, no correlation was found between depression severity (measured with HDRS) and PON 1 concentration.

The contradictory results of these studies may also arise from the fact that both the activity and the expression of paraoxinases can be modulated by various factors. PON transcription is modulated by many factors related to inflammation, oxidative stress and cholesterol levels. Both oxidative stress and immune activation can inhibit the activity and expression of PON1. Decreased PON1 activity may, in turn, increase the oxidative stress (OS) [19]. The effect of fibrates and statins on PON1 expression has been studied the most. These medications are believed to induce PON1 expression [32]. Smoking is associated with decreased PON1 activity. Polyphenols, the Mediterranean diet, oleic acid and physical exercise can increase PON1 activity [19].

Considering the fact that patients in our depression group were slightly older than the healthy controls, we took a closer look at the impact of age on the activity and expression of paraoxinases. Newborns possess half the paraoxonase 1 activity found in adults [32]. From the day of birth, PON1 activity gradually increases throughout the first year of life, when it reaches a plateau, and thereafter PON1 activity decreases with age [32,33]. However, our findings showed no statistically significant correlation between age and expression of any of the analyzed genes in both the study and control groups.

There are very few studies assessing the expression or even activity of paraoxinases (especially PON2 and PON3) in psychiatric patients. Based on our current state of knowledge, it appears that due to the complexity of external, environmental and genetic factors (polymorphism) and the controversy of reported results, the expression of this group of enzymes may not prove to be the most effective biomarker candidate of depression.

There is a certain relationship between the activity of PON1 and its involvement with HDL and the activity of myeloperoxidase (MPO) [19]. When activated by inflammation, macrophages produce MPO, which can oxidize PON, reducing its activity. This, in turn, may deactivate the inhibitory effect of PON1 on myeloperoxidase (MPO), thereby contributing to an increase in the level of oxidative stress (OS) [19,34].

Research data on the role of myeloperoxidase (MPO) in depression seems to be much more consistent. Vaccarino et al. examined 178 pairs of twins, assessing the occurrence of depressive symptoms and the level of inflammatory markers, including MPO [35]. Twin pairs, in which both twins were depressed, had 32% higher MPO values. Among pairs of dizygotic twins in whom only one twin was depressed, those with MDD had 77% higher MPO than their twin without MDD. Such a relationship was not observed in monozygotic twins [35]. Studies indicate that MPO expression at both mRNA and protein levels is increased in depressed patients [36,37]. Similarly, Somani et al. showed increased MPO activity in this group of patients [38]. Our results contradict these findings, since expression of MPO was significantly lower in our patients with depressive disorders than in the control group, both at the mRNA and protein levels. This may be due to the fact that, although participation in the study was not associated with a change in the therapy, our patients had previously received antidepressant treatment.

The chronicity of the inflammatory process in patients with depression should also be taken into account. A sustained reduction in MPO activity decreases inflammation and increases cellular protection [39]. It is possible that treatment can quickly influence the expression of the analyzed enzymes, leading to neuroprotection. In conclusion, prompt initiation of antidepressant treatment is particularly important.

## 5. Limitations

Eligibility for participation in the study was based on the ICD-10 diagnostic criteria and the CIDI questionnaire. A detailed diagnostic interview minimized the risk for inclusion of patients with other groups of mental disorders, notably subjects with a bipolar affective disorder. There is always the possibility of a false positive finding with multiple comparisons. Participation in the study was not related to any change in treatment. Patients had already received antidepressant pharmacotherapy prior to hospitalization as well as at the time of the study. An effect of treatment on the expression of the studied genes cannot be ruled out.

## 6. Conclusions

Our results contradict many studies on enzymes involved in maintaining oxidative balance. Based on our findings, we doubt whether paraoxonases (PON) or myeloperoxidase (MPO) are promising candidates to be accepted as reliable biomarkers of depression; both their activity and expression depend on many factors and may not only be related to depression. However, it is worth noting that, based on our results, the previously described relationship between PON and MPO appears to be confirmed. Clearly, more studies involving larger patient cohorts with greater homogeneity and without confounding factors must be conducted before definitive conclusions can be drawn.

## Figures and Tables

**Table 1:** Characteristics of the study cohort by nosologic group.

Analyzed Trait	Study Group	Statistical Parameters *			
		*M*	*Me*	*SD*	*Min.–Max.*
**Age (years) ^†^**	**Test group**	47.51	51	11.18	18–67
	**Control group**	29.36	26	8.71	20–53
	**Overall**	41.29	44	13.50	18–67
**Number of hospitalizations**	**Test group**	2.01	1	2.00	0–12
**Disease duration (years)**	**Test group**	6.18	4	7.05	1–40
**Number of episodes**	**Test group**	4.56	2	5.33	1–20
**Hamilton Depression Rating Scale (HDRS)**	**Test group**	22.82	23	6.86	1–51

(* Explanations of abbreviations used in result tables: *M*—mean; *Me*—median; *SD*—standard deviation; ^†^ Statistical significance of differences: *p* < 0.0001 for the multifactor generalized linear model fitted, *p* < 0.001 for “by-group” comparison, *p* = 0.528 for “by-gender” comparison).

**Table 2:** Detailed descriptive statistics for gene (mRNA) expression by study group.

Gene	Study Group	Statistical Parameter							
		*M*	*Trim. M*	*Me*	*Q*_1_ *–Q*_3_ *(IQR)*	*SD*	*SE*	95% *CI*	*Min.–Max.*
**PON1 mRNA**	**Test group**	0.572	0.564	0.483	0.446–0.732 (0.286)	0.170	0.012	0.548–0.597	0.363–1.002
	**Control group**	0.558	0.556	0.536	0.479–0.657 (0.178)	0.114	0.011	0.535–0.580	0.327–0.834
	**Overall**	0.567	0.559	0.511	0.451–0.672 (0.221)	0.153	0.009	0.550–0.585	0.327–1.002
**PON2 mRNA**	**Test group**	1.551	1.542	1.559	1.330–1.757 (0.427)	0.324	0.023	1.504–1.596	0.849–2.446
	**Control group**	0.986	0.986	0.988	0.910–1.095 (0.185)	0.126	0.013	0.961–1.011	0.694–1.290
	**Overall**	1.356	1.338	1.327	1.002–1.616 (0.614)	0.382	0.022	1.312–1.400	0.694–2.446
**PON3 mRNA**	**Test group**	0.692	0.680	0.617	0.566–0.830 (0.264)	0.181	0.013	0.666–0.718	0.323–1.226
	**Control group**	0.635	0.632	0.617	0.517–0.762 (0.245)	0.156	0.016	0.604–0.666	0.349–0.977
	**Overall**	0.672	0.664	0.617	0.554–0.802 (0.248)	0.174	0.010	0.652–0.692	0.323–1.226
**MPO mRNA**	**Test group**	**0.305**	**0.307**	**0.338**	**0.231–0.367 (0.136)**	**0.088**	**0.006**	**0.292–0.317**	**0.108–0.513**
	**Control group**	**0.351**	**0.353**	**0.365**	**0.282–0.441 (0.159)**	**0.094**	**0.009**	**0.333–0.370**	**0.162–0.507**
	**Overall**	**0.321**	**0.322**	**0.339**	**0.258–0.378 (0.120)**	**0.093**	**0.005**	**0.310–0.332**	**0.108–0.513**

(PON1—Paraoxonase 1 gene; PON2—Paraoxonase 2 gene; PON3—Paraoxonase 3 gene; MPO—Myeloperoxidase gene; Statistical significance of differences by study groups: PON1 *p* = 0.5895; PON2 *p* = 0.1342; PON3 *p* = 0.2818; **MPO *p* = 0.0014;** All empirical data considering the gene expression had been log transformed before testing the hypotheses. All the models fitted were controlled for age and gender). Bold font indicates statistical significance.

**Table 3:** Detailed descriptive statistics for protein concentrations (ng/mL) by study group.

Gene	Study Group	Statistical Parameter						
		*M*	*Me*	*Q*_1_ *–Q*_3_ *(IQR)*	*SD*	*SE*	95% *CI*	*Min.–Max.*
**PON1 [ng/mL]**	**Test group**	2.208	1.186	1.687–2.830 (1.143)	0.700	0.050	2.108–2.308	1.350–4.010
	**Control group**	2.138	2.088	1.846–2.533 (0.687)	0.454	0.045	2.048–2.228	1.204–3.242
	**Overall**	2.184	1.933	1.700–2.601 (0.901)	0.626	0.037	2.112–2.256	1.204–4.010
**PON2 [ng/mL]**	**Test group**	**5.809**	**5.825**	**4.935–6.623 (1.688)**	**1.194**	**0.087**	**5.639–5.980**	**3.380–9.010**
	**Control group**	**3.803**	**3.980**	**3.599–4.076 (0.477)**	**0.454**	**0.045**	**3.713–3.893**	**1.593–4.797**
	**Overall**	**5.117**	**4.915**	**4.007–6.063 (2.056)**	**1.384**	**0.081**	**4.957–5.277**	**1.593–9.010**
**PON3 [ng/mL]**	**Test group**	2.674	2.643	2.177–3.248 (1.071)	0.709	0.051	2.572–2.775	1.180–4.530
	**Control group**	2.461	2.358	1.979–2.915 (0.936)	0.643	0.064	2.334–2.589	1.294–3.906
	**Overall**	2.600	2.355	2.127–3.109 (0.982)	0.693	0.041	2.520–2.680	1.180–4.530
**MPO [ng/mL]**	**Test group**	**1.116**	**1.126**	**0.807–1.352 (0.545)**	**0.349**	**0.025**	**1.067–1.166**	**0.314–1.943**
	**Control group**	**1.316**	**1.370**	**1.022–1.664 (0.642)**	**0.379**	**0.038**	**1.241–1.391**	**0.562–1.919**
	**Overall**	**1.185**	**1.284**	**0.932–1.405 (0.473)**	**0.371**	**0.022**	**1.142–1.226**	**0.314–1.943**

(PON1—Paraoxonase 1 gene; PON2—Paraoxonase 2 gene; PON3—Paraoxonase 3 gene; MPO—Myeloperoxidase gene; Statistical significance of differences by study groups: PON1 *p* = 0.2274; **PON2 *p* < 0.0001**; PON3 *p* = 0.0363; **MPO *p* = 0.0006;** All the models fitted were controlled for age and gender). Bold font indicates statistical significance.

**Table 4:** Correlation between gene expression and clinical variables. * indicates statistical significance.

			Number of Hospitalizations	Disease Duration (Years)	Number of Episodes	HDRS
**Spearman’s rho**	PON1 [ng/mL]	Correlation Coefficient	−0.026	−0.022	−0.097	−0.057
		Sig. (2-tailed)	0.7244	0.7633	0.1900	0.4385
		N	186	186	186	184
	PON1 mRNA	Correlation Coefficient	0.000	−0.003	−0.076	−0.037
		Sig. (2-tailed)	0.9999	0.9683	0.2997	0.6212
		N	186	186	186	184
	PON2 [ng/mL]	Correlation Coefficient	0.210 *	0.062	0.111	−0.003
		Sig. (2-tailed)	0.0040	0.4003	0.1311	0.9650
		N	186	186	186	184
	PON2 mRNA	Correlation Coefficient	**0** **.** 202 *	0.056	0.103	0.000
		Sig. (2-tailed)	**0.0057**	0.4460	0.1623	0.9983
		N	**186**	186	186	184
	PON3 [ng/mL]	Correlation Coefficient	**−0.033**	−0.047	−0.110	−0.054
		Sig. (2-tailed)	**0.6530**	0.5215	0.1334	0.4707
		N	**186**	186	186	184
	PON3 mRNA	Correlation Coefficient	−0.029	−0.044	−0.112	−0.073
		Sig. (2-tailed)	0.6926	0.5538	0.1277	0.3248
		N	186	186	186	184
	MPO [ng/mL]	Correlation Coefficient	0.039	0.058	0.075	0.057
		Sig. (2-tailed)	0.5954	0.4291	0.3113	0.4404
		N	186	186	186	184
	MPO mRNA	Correlation Coefficient	0.047	0.066	0.076	0.055

**Table 5:** Spearman’s correlations of gene expression with age by study group. * indicates statistical significance.

		PON1 [ng/mL]	PON1 mRNA	PON2 [ng/mL]	PON2 mRNA	PON3 [ng/mL]	PON3 mRNA	MPO [ng/mL]	MPO mRNA
**Test group**	Correlation Coefficient	−0.013	0.001	0.014	0.015	−0.035	−0.036	0.014	0.034
	Sig (2-tailed)	0.8634	0.9887	0.8462	0.8352	0.6350	0.6232	0.8531	0.6431
	N	190	190	190	190	190	190	190	190
**Control group**	Correlation Coefficient	0.002	0.002	−0.044	−0.050	0.143	0.152	−0.009	−0.023
	Sig. (2-tailed)	0.9814	0.9806	0.6659	0.6214	0.1576	0.1327	0.9305	0.8192
	N	99	99	99	99	99	99	99	99
**Overall**	Correlation Coefficient	−0.060	−0.0566	0.462 *	0.463 *	0.074	0.079	−0.133 *	−0.125 *
	Sig. (2-tailed)	0.3059	0.3455	**0.0000**	**0.0000**	0.2119	0.1798	**0.0235**	**0.0340**
	N	289	289	**289**	**289**	289	289	**289**	**289**

## Data Availability

The data that support the findings of this study are available upon request from the corresponding author—M.G.
